# Composite Interpolation-Based Multiscale Fuzzy Entropy and Its Application to Fault Diagnosis of Rolling Bearing

**DOI:** 10.3390/e21030292

**Published:** 2019-03-18

**Authors:** Qingyun Liu, Haiyang Pan, Jinde Zheng, Jinyu Tong, Jiahan Bao

**Affiliations:** 1 School of Mechanical Engineering, Anhui University of Technology, Maanshan 243032, China; 2 Engineering Research Center of Hydraulic Vibration and Control, Ministry of Education, Maanshan 243032, China

**Keywords:** multiscale entropy, multiscale fuzzy entropy, composite interpolation multiscale fuzzy entropy, Laplacian support vector machines, fault diagnosis

## Abstract

Multiscale fuzzy entropy (MFE), as an enhanced multiscale sample entropy (MSE) method, is an effective nonlinear method for measuring the complexity of time series. In this paper, an improved MFE algorithm termed composite interpolation-based multiscale fuzzy entropy (CIMFE) is proposed by using cubic spline interpolation of the time series over different scales to overcome the drawbacks of the coarse-grained MFE process. The proposed CIMFE method is compared with MSE and MFE by analyzing simulation signals and the result indicates that CIMFE is more robust than MSE and MFE in analyzing short time series. Taking this into account, a new fault diagnosis method for rolling bearing is presented by combining CIMFE for feature extraction with Laplacian support vector machine for fault feature classification. Finally, the proposed fault diagnosis method is applied to the experiment data of rolling bearing by comparing with the MSE, MFE and other existing methods, and the recognition rate of the proposed method is 98.71%, 98.71%, 98.71%, 98.71% and 100% under different training samples (5, 10, 15, 20 and 25), which is higher than that of the existing methods.

## 1. Introduction

Rolling bearings are some of the most common mechanical parts in rotating machinery and their running state often affects the performance of the whole machine [1,2]. Therefore, it is of importance to study the incident fault diagnosis method of rolling bearings. Although the linear and stationary signal analysis methods have been widely used for machine monitoring, the vibration signals often present complexity and non-stationarity caused by the variable load operating conditions or nonlinear friction factors. Therefore, the traditional linear and stationary signal analysis methods inevitably have their limitations. With the development of nonlinear dynamic theory, many nonlinear parameters, such as fractal dimension [3,4,5], approximate entropy [6,7], sample entropy [8,9] and multiscale sample entropy [10,11,12] have been used in mechanical fault diagnosis. For example, Yang et al. [4] proposed an intelligent fault diagnosis of rolling element bearings based on support vector machines (SVMs) and fractal dimensions. Han and Pan proposed a fault diagnosis method combined with local mean decomposition (LMD), sample entropy and energy ratio for rolling bearing [8]. Zhang et al. [12] proposed a bearing fault diagnosis method by using multiscale entropy (MSE) and adaptive neuro-fuzzy inference. However, the coarse graining in MSE usually results in the ‘flying wing’ phenomenon at the end with the increase of scale factor. In fact, MSE mainly has the following three defects: (i) the length of each coarse-grained time series divided by the scale factor often is shorter for a larger scale factor, which causes the increase on the MSE entropy deviation with the increase of scale factor. (ii) The process of coarse-grained MSE is regarded as a result of linear interpolation, which has some limitations when analyzing non-stationary and nonlinear data. (iii) The similarity of two vectors defined in sample entropy is based on the step function, which will cause the similarity mutation [13]. Composite multiscale entropy (CMSE) was proposed in [14] to overcome the shortcoming (i) of MSE and in CMSE the obtained information is more accurate and objective. However, the defects (ii) and (iii) still exist. Recently, composite multiscale fuzzy entropy proposed by Zheng et al. [15] can eschew the shortcomings (i) and (iii) of MSE, i.e., the fuzzy entropy was used to replace sample entropy used in MSE and CMSE to avoid the similarity mutation of two compared templates [13]. Nibaldo and Yan have proposed an improved wavelet entropy analysis method and improved dimension entropy method [16,17,18], but the advantages of these methods are not improved methods to solve the above defects, so they can not completely solve the above defects. 

In this paper, the composite interpolation-based fuzzy entropy (CIMFE) is proposed to overcome the drawbacks of MSE. In CIMFE, firstly the composite multiscale and moving average steps are combined to overcome the entropy deviation caused by decreasing data length. Meanwhile, cubic spline interpolation of time series is used to replace the coarse-grained process, which preferably describes the change of nonlinear and non-stationary signals. In addition, fuzzy entropy is utilized to replace sample entropy in MSE to overcome the mutation of similarity measurement. Therefore, CIMFE can overcome the shortcomings of MSE to some degree and has better performance than MSE. Therefore, CIMFE can be used to measure the complexity of mechanical vibration signals.

Next, CIMFE is applied to extract the nonlinear mechanical fault features from vibration signals of rolling bearings. After that an intelligent multi-classifier is needed to achieve an automatic fault mode identification. There are many automatic fault mode identification methods, such as decision tree clustering analysis, gray clustering analysis, fuzzy clustering analysis and neural network, but they lack universality [19]. SVM, as a supervised learning method [20] which requires knowing all class labels of training samples, has been widely used in mechanical fault diagnosis [21,22,23,24]. However, in many places, it is difficult to label all samples. Laplacian support vector machine [25] (LapSVM) is a semi-supervised machine learning method which combines the idea of manifold learning and utilizes the intrinsic manifold structure information from unlabeled samples into the classifier design. LapSVM has been applied to the fault diagnosis of rotating machinery and has achieved good classification results [26]. In this paper, LapSVM is employed to fulfill an intelligent fault diagnosis of rolling bearings. Then a fault diagnosis method for rolling bearings is put forward based on CIMFE and LapSVM. Finally, the proposed method is applied to the analysis of rolling bearing experimental data and the result shows that the proposed method effectively detects the complexity changes of rolling bearing and effectively realizes the fault diagnosis of rolling bearings.

The rest of this paper is organized as follows: Section 2 reviews the MSE and CMSE algorithms and then CIMFE is proposed for complexity measurement of time series. In Section 3, the comparison analysis for 1/*f* noise and Gaussian white noise signal is conducted. Section 4 brings the proposed CIMFE and LapSVM-based fault diagnosis method for rolling bearing and validates the proposed method by analyzing the experimental data of rolling bearings. Finally, Section 5 provides the conclusions.

## 2. Composite Interpolation Multiscale Fuzzy Entropy

### 2.1. MSE and CMSE Methods

The steps of MSE can be simply described as follows [10,11]:

(1) For original data $\left\{X_{i}\}=\{x_{1},x_{2},\ldots ,x_{N}\right\}$ with length *N*, the coarse-grained sequence is established as:
  (1) $$y_{j}(\tau )=\frac{1}{\tau }\sum_{i=(j-1)\tau +1}^{j\tau }x_{i},1\leq j\leq N/\tau$$
  where τ is scale factor. $y_{j}(1)$ is the original time series and when τ>1, $\left\{X_{i}\right\}$ is divided into τ coarse-grained sequence $\left\{y_{j}(\tau )\right\}$ with length [N/τ] (no greater than the positive integer N/τ).
(2) Sample entropy of each coarse-grained sequence $\left\{y_{j}(\tau )\right\}$ are calculated under the same similar tolerance as follows:
  (2) $$MSE(X,\tau ,m,n,r)=SampEn(y^{\tau },m,n,r)$$
  where, *m* is the embedding dimension, *n* is the exponential function gradient parameter, and *r* is the tolerance. Then all obtained entropies can be seen as a function at scale factor τ and this process is called multiscale entropy analysis.

**Figure 1:**
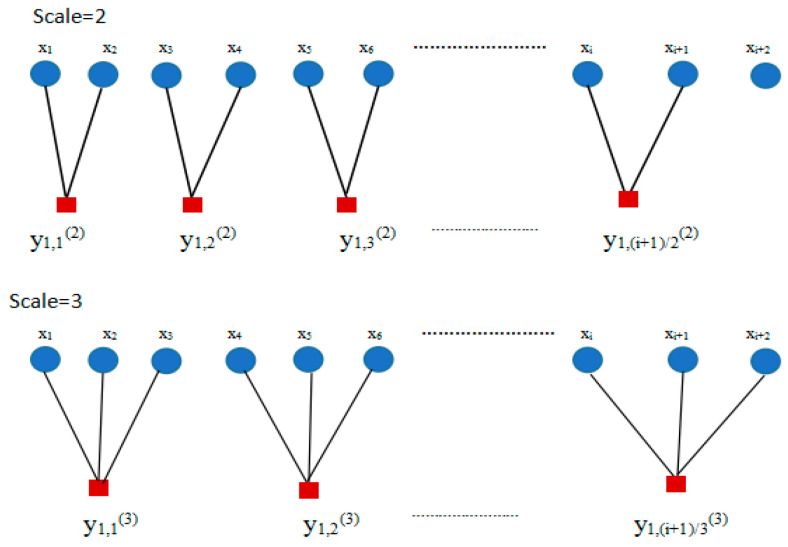
The coarse-grained process when scale factor equals to 2 and 3.

MSE overcomes the defects of sample entropy in single scale for measuring the complexity of time series. However, the coarse-grained process greatly depends on the length of time sequence, which possibly results in the increasing of entropy deviation with the increasing of scale factor in the calculation of MSE. Taking scale factor 2 and 3 as examples and Figure 1 shows the coarse-grained process.

In the coarse-grained process, when τ equals 2 the coarse-grained sequence beginning with the average of $x_{1}$ and $x_{2}$, is solely considered and the time sequence beginning with average of $x_{2}$ and $x_{3}$ is ignored, which also owns the same scale factor 2. Similarly, when τ equals 3, only the coarse-grained sequence beginning with the average of $x_{1}$, $x_{2}$ and $x_{3}$ is used while that beginning with the average of $x_{2}$, $x_{3}$ and $x_{4}$, or $x_{3}$, $x_{4}$ and $x_{5}$ are ignored. Besides, the mean way in original coarse-grained process is seen as a linear interpolation which will cause the non-linearity and non-stationarity of the vibration signal ignored. The steps of CMSE can be simply described as follows [14]:

(1) For the time series {x(i),i=1,2,⋯,N} with data length *N*, the coarse grained series $y_{k}^{\tau }$, $y_{k,j}^{\tau }=\left\{y_{k,1}^{\tau },y_{k,2}^{\tau },\cdots ,y_{k,p}^{\tau }\right\}$ can be calculated as:
  (3) $$y_{k,j}^{\tau }=\frac{1}{\tau }\sum_{i=(j-1)\tau +k}^{j\tau +k-1}x_{i},\;1\leq j\leq N/\tau ,\;\;\;1\leq k\leq \tau$$
(2) For each scale factor τ and k, the SampEns of each coarse grained sequence $y_{k}^{\tau }$ (1≤k≤τ) are calculated and then the average of all the *k* SampEn values for each τ are computed. The CMSE for scale factor τ is obtained by:
  (4) $$CMSE(X,\tau ,m,r)=\frac{1}{\tau }\sum_{j=1}^{\tau }SampEn(y_{j}^{\tau },m,r)$$

**Figure 2:**
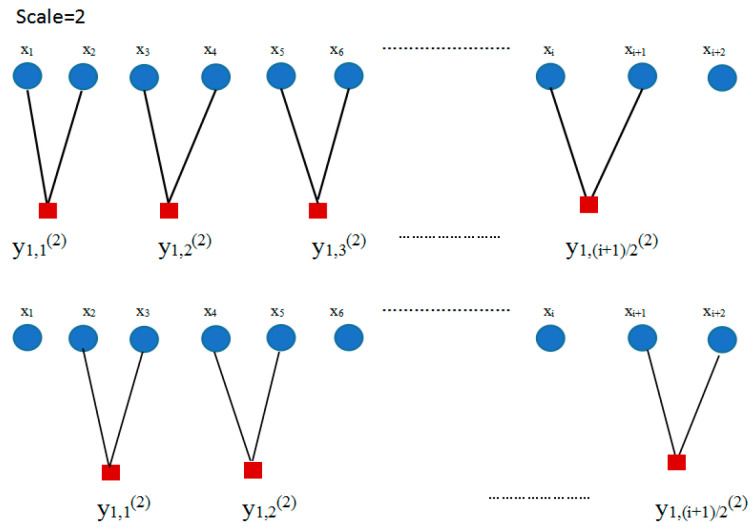
Composite multiscale method with scale factor equal to 2.

The composite multiscale way is displayed in Figure 2 with scale factor equals 2.

### 2.2. Composite Interpolation-Based Multiscale Fuzzy Entropy

In this paper, CIMFE is proposed to solve the deficiencies of MSE. In CIMFE, firstly, fuzzy entropy is employed to replace sample entropy and overcome the mutation of similarity measurement of two vectors. Secondly, the cubic spline method is used to interpolate the coarse-grained time series over different scales for retaining the non-stationarity and nonlinearity of signal. Lastly, the composite multiscale way is used to construct the final entropy values in each scale. The calculation steps of CIMFE are given as follows:

(1) For a given normalized time series {x(i),i=1,2,⋯,N}, the coarse-grained sequence $y_{k,j}^{\tau }=\left\{y_{k,1}^{\tau },y_{k,2}^{\tau },\cdots ,y_{k,p}^{\tau }\right\}$ is expressed as Equation (3).

(2) FuzzyEn of each coarse graining time sequence $y_{k}^{\tau }(1\leq k\leq \tau )$ in each scale factor τ is calculated, and then the mean value of all τ entropies is computed. The CIMFE under this scale factor is described as:

(5) $$CIMFE_{1}(X,\tau ,m,n,r)=\frac{1}{\tau }\sum_{k=1}^{\tau }FuzzyEn(y_{k}^{\tau },m,n,r)$$

where the detailed steps for FuzzyEn calculation can be found in literature [27,28].

**Figure 3:**
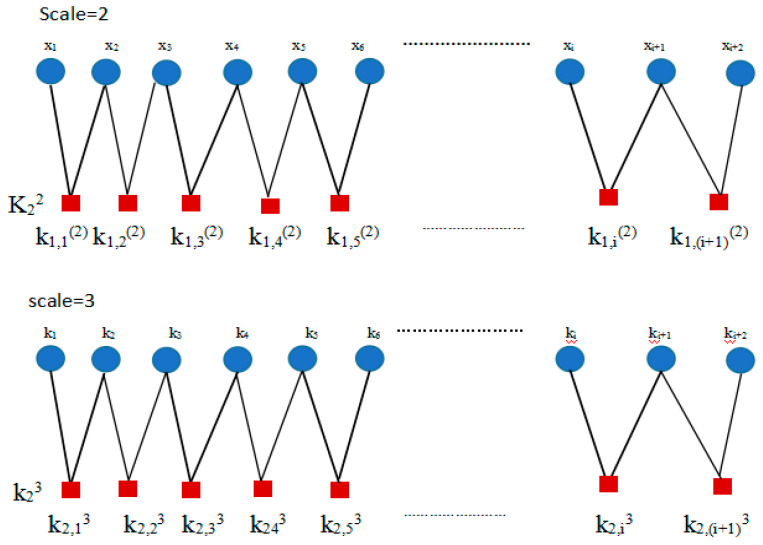
The interpolation coarse graining time series.

(3) For {x(i),i=1,2,⋯,N}, the cubic spline function is applied to interpolate the coarse graining time series over different scales. As shown in Figure 3, when τ equals 1, $k_{1,j}^{1}$ is the original time series $\left\{x_{i}\right\}$. When τ equals 2, $k_{2,j}^{2}$ is the middle point between $x_{i}$ and $x_{i+1}$ (*i* = 1, 2, 3,…) calculated by the cubic spline interpolation function and is given as $k_{2,j}^{2}=\left\{k^{2}(i),1\leq i\leq N-1\right\}$. When τ equals 3, $k_{3,j}^{3}$ is the middle point between $y_{N-2}^{2}$ and $y_{N-1}^{2}$ (*N* = 3,4,5…) calculated by the cubic spline interpolation function and is expressed as $k_{3,j}^{3}=\left\{k^{2}(i),1\leq i\leq N-2\right\}$. The rest can be done in the same ways.

(4) Then FuzzyEn of $k_{p}^{\tau }(1\leq p\leq \tau )$ is calculated and the mean value of all τ FuzzyEn entropies is computed. The CIMFE_2_ under scale factor τ is obtained by:

(6) $$CIMFE_{2}(X,\tau ,m,n,r)=\frac{1}{\tau }\sum_{p=1}^{\tau }FuzzyEn(k_{p}^{\tau },m,n,r)$$

(5) The final CIMFE is defined as:

(7) $$\begin{array}{ll} CIMFE(X,\tau ,m,n,r) & =\frac{1}{2}\sum_{p=1}^{\tau }\left[CIMFE_{1}(X,\tau ,m,n,r\right)\\ & +CIMFE_{2}(X,\tau ,m,n,r)] \end{array}$$

The obtained entropies are expressed as a function of scale factor and this process is called composite interpolation multiscale fuzzy entropy analysis. 

## 3. Comparison Analysis of CIMFE, MSE, CMSE and MFE

### 3.1. Parameter Selection

The calculation of CIMFE is related with data length *N*, embedding dimension *m*, the tolerance *r* and the exponential function gradient parameter *n*. Firstly, with the increase of *m*, more detailed information can be obtained, however, more data length is required $\left(N=10^{m}\sim 30^{m}\right)$. Therefore, we generally set *m* as 2. Secondly, the similar tolerance *r* controls the width of fuzzy function. A smaller *r* results in more statistical information, which will increase the sensitivity of CIMFE to noise. However, a larger *r* will lose some statistical information. Thus, *r* is selected as 0.1~0.25SD (SD is the standard deviation of original data) in this paper, and 0.15SD is used. Thirdly, *n* generally controls the gradient of fuzzy function, and the used fuzzy function will turn into a unit step function when *n* tends to infinity. According to literature [27,28], *n* is set as 2. Lastly, the data length *N* also has some influence on sample entropy and fuzzy entropy, and we keep $N_{\tau }\geq 10^{m}$, where τ is scale factor [14].

### 3.2. Comparison Analysis

**Figure 4:**
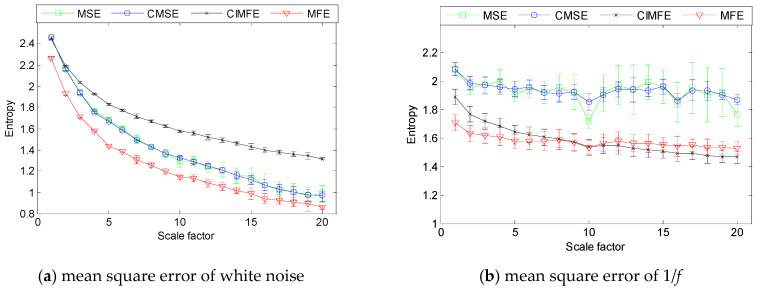
Mean-square curves of different methods about white noise 1/*f* noise: (**a**) white noise and (**b**) 1/*f* noise.

Without loss of generality, we take 20 groups of Gaussian white noises and 1/*f* noises with length 3000 as examples. Their mean-standard deviation curves of MSEs, CMSEs, MFEs and CIMFEs are shown in Figure 4a,b. First of all, from Figure 4, we can find that with the increase of scale factor, the CIMFE curve is relatively smoother and has small fluctuations. Secondly, comparing MSEs, CMSEs and MFEs at each scale factor, CIMFEs fluctuate less slightly, which shows that CIMFEs are more stable than the existing MSE, MFE and CMSE methods. Finally, with the increase of scale factor, the entropies of Gaussian white noise decrease gradually, which shows that compared with 1/*f* noise, the Gaussian white noise signal is simpler and the significant information can be extracted at smaller scales. However, with the increase of scale factor, the entropy curve of 1/*f* noise changes slowly and is basically stable in the vicinity of a constant value. The above analysis indicate that the 1/*f* noise is more complex than white noise and contain important pattern information at all scales, which is consistent with the physical sense of Gaussian white noise and 1/*f* noise signals.

## 4. CIMFE Based Fault Diagnosis Method of Rolling Bearing

### 4.1. The Proposed Fault Diagnosis Method

The above analysis of simulation signals indicates that CIMFE as a new complexity measure method for time series that can get much better performance than the existing methods, i.e., MSE, MFE and CMSE. Thus, CIMFE as the method for fault feature extraction is applied to diagnosis for rolling bearings. At present, monitoring technologies have been widely developed and more fault samples can be collected easily, however it is difficult to mark all the fault classes of the collected samples. Actually, a too small marked samples input into classifiers will result in a worse performance in default recognition. Therefore, it is of significance to promote and improve identification rate by using few marked samples and many unmarked samples [29]. LapSVM, a kind of semi-supervised learning method, can effectively improve classification performance by applying few labeled samples and many unlabeled samples to design the internal structure of the classifier. The procedures of the proposed fault diagnosis method for rolling bearings are given as follows: 

(1) For the given *K* states of rolling bearing, each state has *m_k_* samples and thus the number of whole samples is $M=\sum_{k=1}^{K}m_{k}$.

(2) CIMFE of all *M* samples are calculated with the largest scale factor $\tau_{m}$ and then the CIMFE based feature sets $T^{k}\in R^{m_{k}\times \tau_{\max}}$ can be obtained for the *K* classes (*k* = 1,2,…*K*), where $R^{m_{k}\times \tau_{\max}}$ stands real matrix with row $m_{k}$ and column $\tau_{\max}$. 

(3) For each class, *m_k_* samples are randomly divided into *h* marked sample sets, i.e., $T_{1}^{k}\in R^{h\times \tau_{\max}}$ for training and (*m_k_* − *h*) unmarked sample sets, i.e., $T_{2}^{k}\in R^{(m_{k}-h)\times \tau_{\max}}$ for testing;

(4) The selected sensitive fault feature sets that consists of the first several elements from $T_{1}^{k}\in R^{h\times \tau_{\max}}$ and $T_{2}^{k}\in R^{(m_{k}-h)\times \tau_{\max}}$ are input to the LapSVM-based multi-classifier for fault recognition and the fault locations can be diagnosed according to the outputs of multi-classifier. 

### 4.2. Experiment Data Analysis

Case 1:

In general, the vibration signals, which contain noise signals, express non-stationarity and non-linearity. However, it is a key step to extract fault pattern information from vibration signal in the procedure of fault diagnosis. Both sample entropy and fuzzy entropy have two similar advantages: anti-noise and anti-interference.

Therefore, a new fault diagnosis method for rolling bearings based on CIMFE and LapSVM is proposed in this paper. Firstly, CIMFEs of vibration signals of rolling bearings under different states are calculated and analyzed at different scale factors, if the difference of CIMFE curves of vibration signals of rolling bearings under different states is obvious, they would be used to directly recognize the state of rolling bearings. Otherwise, they would be taken as characteristic vectors and input to LapSVM classifier to conduct the fault recognition for rolling bearings. By using LapSVM, the obtained samples are utilized to train and test the fault classifier, and the diagnosis recognition for rolling bearings are conducted. 

**Figure 5:**
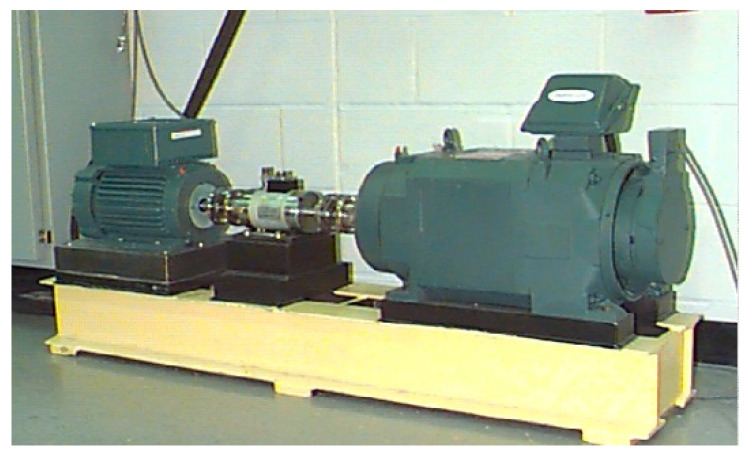
The experimental device.

**Figure 6:**
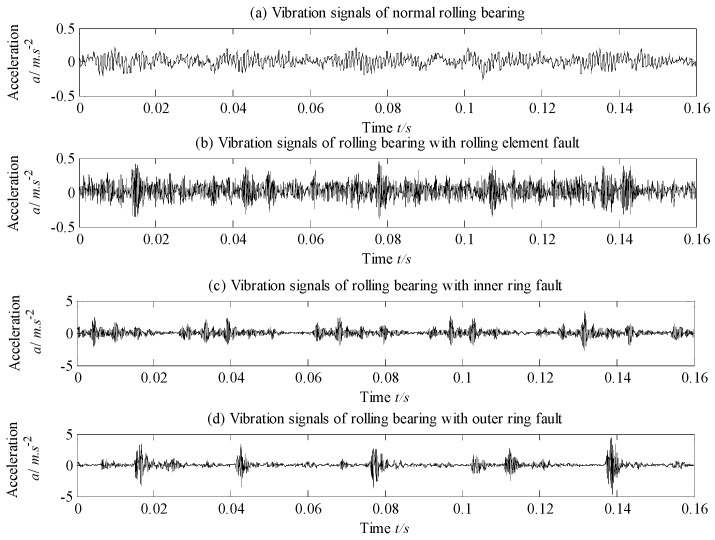
Time-domain vibration signal of rolling bearing under different states: (**a**) vibration signal of normal rolling bearing; (**b**) vibration signal of rolling bearing with ball element fault; (**c**) vibration signal of rolling bearing with inner ring fault and (**d**) vibration signal of rolling bearing with outer ring fault.

The adopted experimental data are derived from the rolling bearing test data supported by Case Western Reserve University in USA [30], and the experimental device is shown in Figure 5. In the test, the tested rolling bearing is 6205-2RS JEM of SKF with fault diameter 0.5334 mm, failure depth 0.2794 mm, speed 1797 r/min and sampling frequency 12 kHz. The vibration signals of rolling bearing with four states, i.e., inner ring fault (IR), outer ring fault (OR), ball element fault (BE) and normal state (Norm) are collected, which are exhibited in Figure 6a–d. As shown in Figure 6, it is difficult to directly distinguish all the states of rolling bearing. 

**Figure 7:**
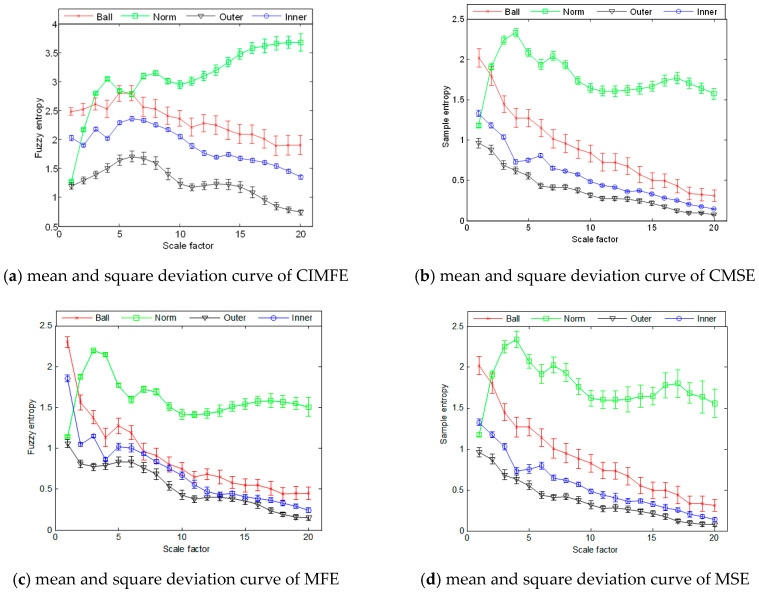
Mean and square deviation cure of different entropy algorithms: (**a**) mean and square deviation curve of CIMFE; (**b**) mean and square deviation curve of CMSE; (**c**) mean and square deviation curve of MFE and (**d**) mean and square deviation curve of MSE.

In addition, 58 samples of rolling bearing vibration signals at each state are selected. The mean and square deviation curves of them is shown in Figure 7a–d, where the data length is set to 2048. Firstly, it can be indicated that when the rolling bearings have different faults, the entropies are different. With the increase of scale factors, MSE, MFE and CMSE curves decrease gradually, and the fluctuation of entropy curves under inner ring fault, outer ring fault and rolling element fault conditions are relatively consistent. However, with the increase of scale factor, the fluctuations of CIMFE curves are smaller and smoother than that of other methods, which is beneficial to improve and promote the recognition performance. Secondly, the vibration signal of rolling bearings at normal state are random and irregular, hence the arising tendency of its entropy curve is expressed with the increase of scale factor, which demonstrates that the normal bearing signals include crucial information both in the lower scales and higher scales. 

Eventually, due to the working conditions of rolling bearings where the ball element rolls and the inner ring rotates with the rotational motion of axis and the outer ring is fixed on the bearing pedestal. Those result in that the entropies of the rolling bearing vibration signals with ball element faults is larger than that with inner ring faults, which is also larger than that with outer ring faults. 

**Figure 8:**
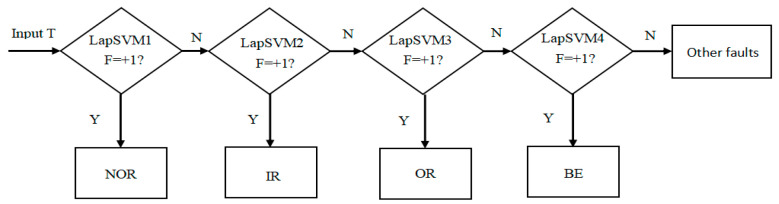
LapSVM based multi-fault classifier.

Case 2:

In order to effectively conduct an intelligent and efficient fault recognization of rolling bearing, 58 samples of each state i.e., Norm, IR, OR and BE, are divided into 25 marked samples and 33 unmarked samples and then input the LapSVM classifier where the radial basis function is selected as kernel function whose parameter is set to 0.35. Based on the partial two binary tree method, LapSVM multi-fault classifier is established, which is exhibited in Figure 8 and Table 1 where LapSVM1, LapSVM2, LapSVM3, LapSVM4 represent the normal state, inner ring fault, outer ring fault and ball element fault, respectively. 

**Figure 9:**
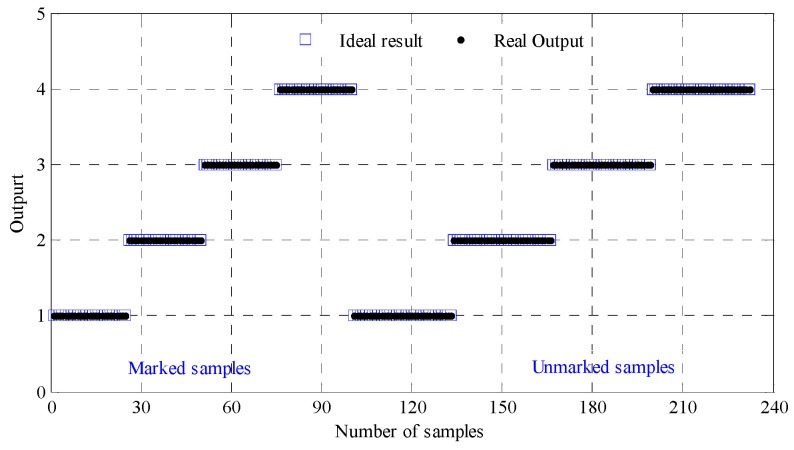
Output results of the LapSVM-based multi-classifier of test samples.

Meanwhile the sample feature vector *T* is input into LapSVM fault classifier when classification test is conducted. The sample outputs are shown in Figure 9, where all the samples are effectively identified, hence the recognition rate is 100%.

**Figure 10:**
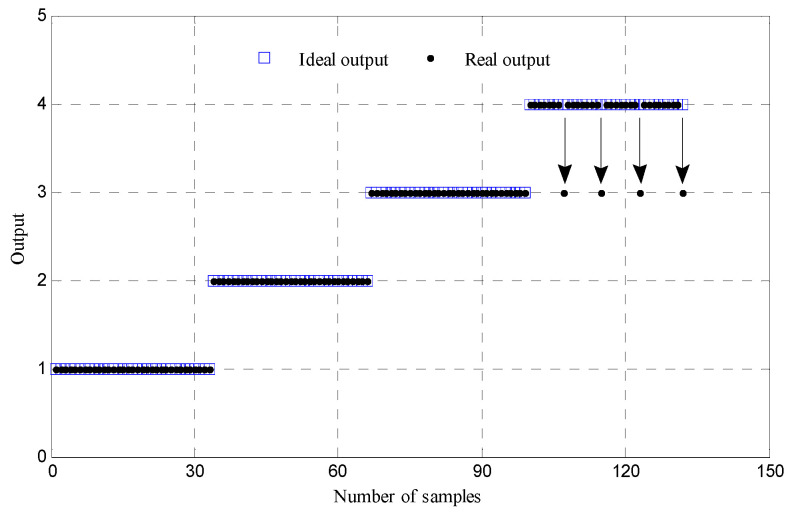
Output results of the SVM based multi-classifier of test samples.

Next, the above obtained 58 groups of CIMFE of four states will also be input to SVM classifier to implement fault recognition for rolling bearing. As before, 25 × 4 groups at four states are taken as marked samples and 32 × 4 groups under four states are regarded as unmarked samples all the samples are input to SVM classifier. Hence the outputs are shown in Table 2, where the LibSVM program is utilized. Gaussian radial basis function is selected as kernel function of SVM. Meanwhile, based on particle swarm optimization algorithm [31], the penalty parameter *c* and kernel function parameter *g* are determined and optimized. As shown in Table 2 and Figure 10, the recognition rate is 96.97% in which four samples with ball element fault are wrongly treated as that with outer ring fault in SVM3, and four samples with ball element fault are not recognized in SVM4. Eventually, comparing Table 1 and Table 2, when much unmarked samples are adopted, the fault type of rolling bearing can be effectively classified by LapSVM, which indicates that the proposed method has a better recognition performance on fault diagnosis of rolling bearing.

Case 3

To further illustrate the superiority of the proposed method and the influence of the number of marked samples on recognition performance, comparisons of CIMFE with MSE, MFE and CMSE, or LapSVM with SVM are conducted. Firstly, to illustrate the superiority of CIMFE, MSE, CMSE and MFE are also applied to extract feature information of vibration signals of rolling bearings. Then MSEs, CMSEs, MFEs and CIMFEs are taken as sensitive feature sets and respectively input to LapSVM classifier with the same parameters for state recognition of rolling bearings. When the number of marked samples is set to 5, 10, 15, 20 and 25 separately, the recognition results are illustrated in Table 3. As shown in Table 3, when the number of marked samples is determined, the recognition rate of the MSE and LapSVM-based methods are different from that of other methods which are almost a same constant, i.e., when the number of marked samples is fifteen, the recognition rate of the MSE and LapSVM-based method is 97.41% and those of the other methods are 98.71%. Secondly, with the increase of the number of marked samples, the identification rate of all methods fluctuates slowly and almost tends to be constant, however, the identification rates of the MSE and LapSVM-based method are always smaller than those of other methods. The above analysis indicates that compared to MSE, MFE and CMSE, CIMFE has a better performance when extracting fault features of rolling bearings. In particular, when the number of marked samples is 25, the recognition rate of the proposed method can reach 100%. 

Next, to illustrate the superiority of LapSVM to SVM, the above entropies of MSE, MFE, CMSE and CIMFE are also input to SVM to conduct the default recognition of rolling bearing and the recognition results are shown in Table 4 where the number of training samples is set to 5, 10, 15, 20 and 25 respectively. As shown in Table 4, when the number of trained samples is determined, the recognition rate of all the SVM-based methods vary. With the increase of the number of trained samples, the recognition rate of all the SVM-based methods fluctuate slightly and in particular, the recognition rate of the CIMFE and SVM-based methods for fault recognition of rolling bearing is always higher than that of the other methods using MSE, MFE and CMSE to extract fault features of rolling bearings. The above analysis indicate that the number of marked samples has some influence on recognition performance. As a measuring complexity method for time series, CIMFE is superiority to MSE, MFE and CMSE. Finally, comparing Table 3 and Table 4, when the method for extracting fault feature is determined, the recognition rate of the LapSVM-based methods has little fluctuation and is always higher than that of the SVM-based methods. By using both SVM and LapSVM to conduct the fault recognition of rolling bearings, the CIMFE-based methods always obtain a better recognition rate. In particular, when the number of marked samples is suitable, the recognition rate of the proposed method can reach 100% which can be seen in Table 3. The comparison analysis shown in Table 3 and Table 4, validate the superiority of the proposed method for the fault diagnosis of rolling bearing. 

## 5. Conclusions

In this paper, a new method for measuring complexity of time series named CIMFE is proposed. The influence of the main parameters i.e., similar tolerance and data length on CIMFEs are researched by analyzing the simulation signals i.e., 1/*f* noised and Gaussian white noise signal. The results suggest that compared to existing entropy algorithms, i.e., MSE, MFE and CMSE, CIMFE embodies more stability and consistency. Meanwhile, the fluctuations of CIMFE curves are smaller and smoother than those of other methods. In addition, combing CIMFE and LapSVM, a new fault diagnosis method for rolling bearings is proposed and applied to analyzing rolling bearing experimental data. The recognition rates of the proposed CIMFE and LapSVM method are 98.71%, 98.71%, 98.71%, 98.71% and 100% under different training samples (5, 10, 15, 20 and 25), which is higher than that of the existing methods, i.e., MSE and LapSVM, CMSE and LapSVM, MFE and LapSVM. At the same time, the recognition rate of the CIMFE and LapSVM method is higher than that of CIMFE and SVM method under different training samples (5, 10, 15, 20 and 25). Now, the entropy theories have not been widely used on extracting feature of mechanical vibration signal. In this paper, we tried to apply the proposal into practice and both simulation and experimental data analysis were conducted. The following work will be emphasized on the cost-time decrease of the calculation, further optimization of the proposed methods and application on the on-line predicting fault.

## Figures and Tables

**Table 1:** Output results of LapSVM classifier.

Samples	State	LapSVM1	LapSVM2	LapSVM3	LapSVM4	Result
T_1_~T_58_	Norm	+1(58)				Norm
T_59_~T_116_	IR	−1(58)	+1(58)			IR
T_117_~T_174_	OR	−1(58)	−1(58)	+1(58)		OR
T_175_~T_232_	BE	−1(58)	−1(58)	−1(58)	+1(58)	BE

**Table 2:** Output results of SVM.

Samples	State	SVM1	SVM2	SVM3	SVM4	Result
T_1_~T_33_	Norm	+1(33)				Norm
T_34_~T_66_	IR	−1(33)	+1(33)			IR
T_67_~T_99_	OR	−1(33)	−1(33)	+1(37)		OR
T_100_~T_132_	BE	−1(33)	−1(33)	−1(29)	+1(29)	BE

**Table 3:** Recognition rate of the methods by using LapSVM (%).

Number of Marked Samples/Method	5	10	15	20	25
MSE	97.41	97.84	97.41	97.84	98.28
CMSE	98.71	98.71	98.71	98.71	98.71
MFE	98.71	99.14	98.71	98.71	98.71
CIMFE	98.71	98.71	98.71	98.71	100

**Table 4:** Recognition rate of the methods by using SVM (%).

Method/Number of Training Samples	5	10	15	20	25
MSE	96.70	96.35	95.93	95.39	96.21
CMSE	96.70	96.75	95.93	95.39	96.21
MFE	97.17	96.88	96.51	96.05	96.97
CIMFE	98.11	97.91	96.67	97.37	98.48
